# Artefacts of Change: The Disruptive Nature of Humanoid Robots Beyond Classificatory Concerns

**DOI:** 10.1007/s11948-025-00532-5

**Published:** 2025-03-28

**Authors:** Cindy Friedman

**Affiliations:** Department of Philosophy and Religious Studies, Utrecht University, Janskerkhof 13, Utrecht, 3512 BL The Netherlands

**Keywords:** Humanoid robot, Social robot, Human-robot interaction, Disruptive technology

## Abstract

One characteristic of socially disruptive technologies is that they have the potential to cause uncertainty about the application conditions of a concept i.e., they are conceptually disruptive. Humanoid robots have done just this, as evidenced by discussions about whether, and under what conditions, humanoid robots could be classified as, for example, moral agents, moral patients, or legal and/or moral persons. This paper frames the disruptive effect of humanoid robots differently by taking the discussion beyond that of classificatory concerns. It does so by showing that humanoid robots are socially disruptive because they also transform how we experience and understand the world. Through inviting us to relate to a technological artefact as if it is human, humanoid robots have a profound impact upon the way in which we relate to different elements of our world. Specifically, I focus on three types of human relational experiences, and how the norms that surround them may be transformed by humanoid robots: (1) human-technology relations; (2) human-human relations; and (3) human-self relations. Anticipating the ways in which humanoid robots may change society is important given that once a technology is entrenched, it is difficult to counteract negative impacts. Therefore, we should try to anticipate them while we can still do something to prevent them. Since humanoid robots are currently relatively rudimentary, yet there is incentive to invest more in their development, it is now a good time to think carefully about how this technology may impact us.

## Introduction

The human fascination with creating artefacts in our image has been with us since the dawn of humankind^1^. However, with the advent of humanoid robotics, we are now seeing creations that do more than tell a story about how we see ourselves: they are also forcing us to face some challenging philosophical questions. Being at the source of these challenging questions constitutes humanoid robots as a type of socially disruptive technology (van der Poel et al., 2023). This is because, in grappling with these questions, it becomes clear that they have the potential to transform our society in different ways. One characteristic of socially disruptive technologies is that they have the potential to cause uncertainty about the application conditions of a concept i.e., they are *conceptually disruptive* (Hopster, 2021; Löhr, 2021, 2022; van der Poel et al., 2023). Humanoid robots have done just this: there is a lot of uncertainty surrounding discussions about whether, and under what conditions, concepts such as moral agency, moral patiency, or personhood could be applied to robots (Friedman, 2020; Gunkel, 2018; Strasser, 2022; Wareham, 2021). Although an interesting and pertinent discussion, this paper seeks to frame the disruptive effect of humanoid robots in a different way. By employing post-phenomenology as one of its take-off points, this paper takes the disruptive nature of humanoid robots beyond that of classificatory concerns by showing that they transform and, thereby, potentially also disrupt how we experience and understand the world. Doing so allows for us to take a broader perspective on the disruptive nature of humanoid robots. This is because while discussion about the conceptual disruptiveness of humanoid robots may impact how we relate to and interact with humanoid robots themselves, taking the discussion beyond conceptual disruption - the way in which this paper suggests - provides a framework within which to analyse how humanoid robots impact *us* and the way in which we relate to other elements of our worlds more broadly. Such an analysis contributes to laying the groundwork for critically reflecting upon whether the societal changes brought about by humanoid robots are desirable. If not, we still have time to revise how we are developing and deploying the technology before it is deeply entrenched in society.

In this paper, my focus is specifically upon the potential impacts of humanoid robots upon human relational experiences. My focus is on *human relational experiences*, given that humanoid robots are specifically designed so as to elicit certain human relational responses from us. Through inviting us to relate to a technological artefact as if it is human, humanoid robots have a profound impact upon the way in which we relate to different elements of our world. Specifically, I focus on three types of human relational experiences, and how the norms that surround them may be transformed by humanoid robots: (1) human-technology relations; (2) human-human relations; and (3) human-self relations.

The paper will proceed as follows: “What are Humanoid Robots?” provides a brief discussion of what humanoid robots are, the nature of our interactions with them, and why I focus upon humanoid robots in particular, as opposed to non-humanoid social robots (or other robots in general); “Humanoid Robots as a Conceptually Disruptive Technology” provides a brief overview of the current debate surrounding the conceptual disruptiveness of humanoid robots, and introduces my reasoning for why and how we should go beyond this debate. Seeking to go beyond conceptual disruption, “Going beyond Conceptual Disruption: Three Ways in which Humanoid Robots May Transform Our Own Human Relational Experiences” discusses three distinct ways in which humanoid robots also disrupt how we experience and relate to the world, specifically by considering how they may transform the norms that surround three types of human relational experiences: firstly, the way in which we relate to technology (“Human-Technology Relations”); secondly, the way in which we relate to other human beings (“Human-Human Relations”); and, lastly, the way in which we relate to ourselves i.e., our human self-understanding (“Human-Self Relations”). “Conclusion” provides a brief conclusion.

## What are Humanoid Robots?

Humanoid robots are robots that are designed and created to replicate human beings both in the way they look, and in the way they simulate human behaviour (Nyholm, 2020). Although humanoid robots are a staple of science fiction, I am here interested in real-world humanoid robots and the philosophical questions they raise, as opposed to more hypothetical questions that arise in relation to the robots we see in books and movies.

Current real-world examples of humanoid robots include Nadine, developed by the MiraLab in Geneva^2^; Hiroshi Ishiguro’s robot, Erica^3^, as well as the robotic replica of Ishiguro himself^4^; and the famous robot Sophia, created by Hanson Robotics^5^. Sophia is clearly modelled based on the appearance of a human being, where the face in particular is made to be as expressive and as humanlike as possible. Moreover, the AI upon which she operates allows for people to socially interact with her, similarly to the way in which they may interact with another human being. This is because Sophia is able to give unique verbal responses in different social contexts, and can also “recognize human faces, see emotional expressions, and recognize various hand gestures” (Hanson Robotics, 2022). Her movement is also human-like in the sense that she can control her “hands, gaze, and locomotion strategy” (ibid.).

Many humanoid robots– such as Sophia– are, therefore, a type of social robot, meaning that one of the intentions behind their development is for them to engage us in human-like relations (Breazeal, 2004). To encourage this engagement, social roboticists draw upon the tendency that human beings have to anthropomorphise, i.e., to attribute human-like characteristics to that which is not human (Damiano & Damouchel, 2018). Research shows that people perceive computers and virtual characters as social actors (Moon & Nass, 2000), and the embodiment and physical movement of robots amplify this perception (cf. Darling, 2016).

Social robots take on many shapes and forms. The robot, Paro, for example, is a social robot that has the form of a baby seal^6^. Even where social robots have non-humanoid forms, there is a tendency for people to anthropomorphise them and form social and emotional bonds with them (Turkle, 2006). However, research has shown that it is the interplay between human-like behaviour, coupled with a distinctly human-like appearance, that causes people to relate to humanoid robots in a more *realistically* human-like way. This is because when a robot looks human and simulates human-like behaviour, our brains are tricked into interpreting this behaviour as if it had been generated by a human being (Sandini & Sciutti, 2018). As such, although we may relate to other social robots in human-like ways due to the tendency to anthropomorphise, humanoid robots draw on this tendency much more strongly. We, therefore, have the tendency to experience much more realistic social relations with them^7^.

It is this tendency to relate to humanoid robots in social and emotional ways, yet also being acutely aware that it is a technological artefact, that is at the foundation for why humanoid robots have the potential to impact our human relational experiences. This will become clearer in the sections that follow.

Although some people do respond to current humanoid robots as if they are human, it must be noted that we are a far way from seeing robots that would actually be mistaken for human beings, given that the humanoid robots that we have today are fairly rudimentary (see Nyholm, 2020, pp. 1–3; Ackerman, 2022). There is, however, incentive to advance their development, such as in the context of care robots for the elderly (Telving, 2022; see also Waytz, 2014). Moreover, given the progress we have already seen, it is not difficult to imagine a future in which these robots do become more convincingly human-like. As already noted, it is a good idea to have discussions about the ethics of new and emerging technologies before those technologies are widely introduced into society, and not only afterwards, where interventions may be hard to make (Collingridge, 1980; see also van de Poel, 2016; Kudina & Verbeek, 2019).

## Humanoid Robots as a Conceptually Disruptive Technology

Much of robot ethics literature is concerned with the conceptual disruptiveness of humanoid robots. Conceptually disruptive technologies are technologies that cause uncertainty about the application conditions of a concept (Löhr, 2022). In other words, a lot of robot ethics literature is concerned with how we may *classify* humanoid robots. Some suggestions include classifying robots as tools (Bryson, 2009; cf. Gunkel, 2023); persons (Wareham, 2021; Jecker et al., 2022); moral subjects (Coeckelbergh, 2010; Danaher, 2019); and legal subjects (Turner, 2019; Gunkel, 2023). Within these discussions, there is uncertainty about under which conditions these concepts may be applied. Do we take a properties-based approach? I.e., do we consider whether robots have the relevant mental capacities (such as consciousness or sentience) to be considered persons (Mosakas, 2021; Müller, 2021)? Do we consider the ways in which we perceive and relate to these robots? I.e., do we classify humanoid robots as persons because we sometimes have the tendency to relate to them as being one of us (Coeckelbergh, 2010; Gunkel, 2018)? Or, do we take both properties and relationality into account (Nyholm & Friedman, 2025 forthcoming)? Therefore, the discussion about humanoid robots as a socially disruptive technology has mostly related to how and why these robots are *conceptually* disruptive (Löhr, 2022): do the concepts of tool, person, moral subject and/or legal subject apply to humanoid robots? Why or why not? And under which conditions could they or could they not apply?

Naturally, debates about how we may classify humanoid robots, have led to related discussions about how we should treat them (Gerdes, 2016; Darling, 2016; Friedman, 2020) and whether we should even consider granting them some type of rights (Gunkel, 2018). Some argue that our tendency to anthropomorphise robots, and relate to them as social entities (see De Graaf, 2016), means that we should treat robots well and grant them legal protection from harmful treatment (Darling, 2016; Gunkel, 2018; Friedman, 2023; see also Turner, 2019; Gellers, 2021; Smith, 2021; Mamak, 2022). Others, however, are sceptical about the moral treatment of robots, arguing that there is no ethical reason for why robots warrant any moral treatment and rights given that they are tools that lack moral status (see Müller, 2021; Sweeney, 2021; see also Birhane & van Dijk, 2020).

As such, when considering the current debate about humanoid robots as a socially disruptive technology, much of the debate is dedicated to their *conceptual* disruptiveness and the impact this disruption may have upon how we relate to and interact with *them*.

However, in order to gain a fuller understanding of how humanoid robots are socially disruptive, we must go beyond thinking about the ways in which humanoid robots are conceptually disruptive, and also think about the ways in which the technology impacts *us*, and how we experience and relate to other elements of our world. One way in which to do this is to consider a post-phenomenological approach, which analyses the ways in which different technologies shape relations between human beings and the world. Using post-phenomenology as a take-off point, the following sections show how humanoid robots are not only conceptually disruptive, but also disruptive in-so-far as they transform how we experience and relate to other elements of our world.

## Going Beyond Conceptual Disruption: Three Ways in Which Humanoid Robots May Transform Our Own Human Relational Experiences

Post-phenomenology is a philosophical framework that analyses the ways in which different technologies shape relations between human beings and the world. Thus, technologies are not seen as being merely functional and instrumental objects but, rather as *mediators* of human experiences. For example, looking at and interpreting the time on a wristwatch transforms how we experience and encounter the world, via our experience with the technology. And eyeglasses, for example, also impact how we experience and encounter the world as they become a part of our perceptual experience (Rosenberger & Verbeek, 2015).

Post-phenomenology can be applied to socially disruptive technologies to help us think about how technologies disrupt our *human experiences*. It, therefore, serves as an ideal take-off point to consider how humanoid robots are more than just conceptually disruptive, but also disruptive in-so-far as they transform how we experience and relate to the world around us.

In the following sections, I discuss three ways in which humanoid robots may transform the way in which we experience and relate to the world: firstly, the way in which we relate to technology; secondly, the way in which we relate to other human beings; and, thirdly, the way in which we relate to ourselves.

### Human-Technology Relations

Humanoid robots are a conceptually disruptive technology because there is uncertainty about their ontological classification: they occupy a liminal space wherein they are more than a tool, yet less than human. This, as noted above, impacts how, and whether, we may apply other concepts to them such as moral or legal subjecthood. This is a well-established debate and, therefore, we may take this debate a step further by considering the *impacts* of this uncertainty. One such impact is upon *human-technology relations*.

When thinking about moral relations with technology, the most widely held and traditional view is an instrumentalist one (Bryson, 2009; Gunkel, 2018, 2023). According to this view, technology is, and should only be viewed as, a mere tool to be utilised to achieve our own ends. This view, however, has been challenged.

Post-phenomenology challenges an instrumental approach to technology by positing that technology is not just an instrumental object or tool, but a mediator between humans and the world (Ihde, 1995; Rosenberger & Verbeek, 2015). In different ways, we experience the world through using technologies which, in turn, impacts how we view and relate to the world around us (Ihde, 1990; Rosenberger & Verbeek, 2015).

However, our perception of humanoid robots as being more than a tool but less than human, challenges an instrumentalist view of technology even further. It opens up the possibility for a technology to go beyond being a mediator, and even be considered an end in itself.

The way in which we perceive humanoid robots, impacts how we morally relate to them. In viewing them as more than a tool, we often feel morally obligated to consequently treat them as such. I.e., we may feel inclined to want to treat them morally well (Carpenter, 2016; Darling, 2021). This raises the question of whether robots could be moral subjects. Many argue that they can, and/or should be (Coeckelbergh, 2010; Darling, 2016; Gunkel, 2018; Danaher, 2019; Nyholm, 2020).

How does this impact human-technology relations on a broader scale? It challenges the longstanding instrumentalist perception of technology by opening up the possibility for a technology to be an end in itself i.e., that a technology can be more than a tool to be used to achieve our own ends, but can be an end in and of itself, thus warranting moral treatment.

As Hall (2001) notes: “we have never considered ourselves to have moral duties to our machines”. We may have a duty not to damage or destroy machines in the sense that they are valuable pieces of property. However, this would be for the sake of the owner of said property, and not because it is considered a moral subject with moral standing (Müller, 2021) i.e., it has instrumental value. Thus, the way in which we normally morally relate to technology is challenged.

On an even broader scale, the consideration of robot moral status opens the opportunity for thinking about the limitations of existing moral and legal systems (Gunkel, 2023). Debates about robot rights naturally follow from those concerned about robot moral status (Asaro, 2012). If there is the inclination to want to treat humanoid robots well, we may consider granting them protection in the form of rights (see e.g., Coeckelbergh, 2010; Gunkel, 2018; Friedman, 2023). Scholarly debate about robot rights contributes to “ongoing efforts to test, validate, and even revise the limits of our moral and legal systems” (Gunkel, 2023, p. 5). Should these systems change, there may be a broad scale change to how we relate to technologies in general. Moral and legal systems may regulate the way in which we treat and interact with technologies in a more systematic way.

### Human-Human Relations

Due to viewing and relating to humanoid robots as if they are a “someone” and not only a “something” (Gunkel, 2023), there is very real potential for people to relate to, and bond with, humanoid robots in emotional ways: we empathise with them (Riek et al., 2009), and show love and care towards them (Turkle, 2006).

The possibility of bonding with these robots in such a way has prompted discussion about the ethical implications of bonding with robots. These discussions point to another way in which humanoid robots may transform the norms that surround some human relational experiences. This disruption may occur in at least two ways: firstly, our relations with other people may be disrupted in the sense that robotic relations may crowd out human relations i.e., social norms are disrupted: when we would normally socially interact with another human, we may interact with a robot. Secondly, the way we morally conduct ourselves within human-human relations may be impacted i.e., moral norms are disrupted. Let us begin by considering the former.

#### Crowding Out Human Relations

Some researchers have voiced their concern about robotic relations crowding out human relations (Turkle, 2006; Danaher, 2019; Nyholm & Frank 2019; Friedman, 2022). This primarily refers to isolated instances wherein individual people may interact with a humanoid robot on a regular basis, and for various reasons, therefore interact much less with other people (Friedman, 2022). Some reasons put forward for why people may prefer robotic companionship over human companionship are that: robots could allow people to engage in interactions that may be frowned upon in the context of human-human relations (e.g., in relation to certain sexual fantasies) (cf. Strikwerda, 2017); provide a safe space for social interaction, should people have social anxiety (Levy, 2007; McArthur, 2017) or be differently abled (cf. Vallor, 2011). Levy (2007) even argues that robots could provide more satisfactory companionship because they can be customised, thus allowing us to satisfy our own preferences within an “ideal” relationship i.e., we could have our perfect partner.

Relations with humans may thereby be disrupted in the sense that people may be less likely to want to interact with other people: they “may be more comfortable within the confines of their robotic relationships because the technology bestows upon them pleasurable benefits that they otherwise would have to exercise their moral agency to receive i.e., interact with other people, and take part in modern civilisation” (Friedman, 2022, p. 530; cf. Danaher, 2019).

Perhaps the very concept of being in a meaningful relationship may change such that the concept becomes more superficial and less multidimensional? This may especially be the case in the context of customisable robots, that could be our “perfect” partners. Could our understanding of being in a relationship eventually amount to simply seeking out preference satisfaction, thereby impacting how and why we seek out *human* companionship? And, since we cannot attain perfect preference satisfaction from another person, this could be another reason why people may seek out human companionship less and less.

We are already seeing something like this occur in Japan with what is termed as *otaku*: “a generation of geeks who have grown up through 20 years of economic stagnation and have chosen to tune out and immerse themselves in their own fantasy worlds” (Rani, 2013). In immersing themselves in their fantasy worlds, many otaku men have shown less interest in starting relationships with human romantic partners, due to the possibility of rather having a “virtual girlfriend” (ibid.; cf Nyholm, 2020). A man named Akihiko Kondo even went so far as to marry his holographic girlfriend in 2018 (Ueno & Dooley, 2022).

More than just acknowledging that this disruption of social norms is a possibility, it should also be noted that this disruption is particularly concerning, given how valuable human-human interaction is. The crowding out of human-human relationships due to forming relationships with robots may, therefore, be concerning. This captures the concern that authors such as Parks (2021), Robert and Linda Sparrow (2006), and Amanda and Noel Sharkey (2012) voice in the context of care robots; that Sven Nyholm and Lily Frank (2019) voice in the context of sex robots; and that Turkle (2011) expresses more generally through her phrase “the robotic moment” i.e., the notion that robots may cause social isolation because they only give an illusion of companionship.

Although these authors have primarily written about social robots in general, many humanoid robots are a type of social robot. Therefore, the same concerns may arise in the context of interacting with humanoid robots in particular. However, more than just being socially disruptive by disrupting the social norms of interacting with people in certain contexts, humanoid robots may also disrupt the moral norm of how we should conduct ourselves within human-human relationships.

#### Moral Conduct Within Human-Human Relations

More than just affecting the very possibility of relating to other people, humanoid robots may also change the way in which we conduct ourselves within human-human relations. Specifically, they may affect the way in which we morally relate to other people, thereby altering certain human relational moral norms. I put forward two instances in which this may occur. Firstly, through bonding with a robot, we may not be able to develop certain relational moral skills, which we would normally develop within human-human relationships. Secondly, the way in which we conduct ourselves in these “bonding” relations with robots, may also impact the way in which we morally conduct ourselves within human-human relationships i.e., how we treat other people.

Firstly, the very possibility of bonding with a robot is socially and morally transformative because, in the context of this human-robot relationship, we may not have ample opportunity to develop certain relational moral skills. As such, moral norms may be altered with regard to how we then go on to relate to other people.

Considering the context of emotionally bonding with, and loving, robots, some argue that robots can be deemed the “perfect” partner. Levy (2007) advocates this view, since there would be the possibility to specify the features of robotic companions (both their appearance and simulated behaviour), and these robots could be preprogrammed to fulfil our every need (Sullins, 2012). For example, the sex robot Roxxxy has different “personality” settings, which satisfy different people’s preferences (Russon, 2017). Moreover, they would be ever faithful, and never leave.

This view, however, has been met with some opposition. For example, Evans (2010), argues that a relationship with the perfect robotic partner– one that would be unwaveringly faithful and never leave you– would be unsatisfactory due to our “desire to be desired”. In other words, we want a partner who chooses to be with us, despite having the freedom to leave. A robot could not fulfil this desire, due to lack of free will. Sven Nyholm and Lily Frank (2017) make a similar claim: if one categorisation of love pertains to making an active commitment to one’s partner, then robots cannot satisfy this commitment if one understands making a commitment in terms of making a free choice.

Although some may be sceptical about robots being “perfect” partners, we may still wonder about the ramifications of interacting with these robotic companions in intimate ways: could the way we relate to other people be affected? Should we become used to interacting with these “perfect” robotic companions, the way in which we relate to other people may be negatively impacted. As noted by Sullins (2012, p. 16) we may “grow less and less tolerant of human relations”, as we may lose our “tolerance to deal with others who are not preprogrammed to serve our every need”. If this occurs, we may also be concerned about what Vallor (2015) terms “moral deskilling”. Vallor (2015, p. 109) writes:

moral skills appear just as vulnerable to disruption or devaluation by technology-driven shifts in human practices as are professional or artisanal skills such as machining, shoemaking, or gardening. This is because moral skills are typically acquired in specific practices which, under the right conditions and with sufficient opportunity for repetition, foster the cultivation of practical wisdom and moral habituation that jointly constitute genuine virtue.

In the context of robot companionship, one virtue that we may have less opportunity to cultivate could be patience. In human-human relations, we cultivate the virtue of patience by way of being understanding and tolerant of people’s imperfections. However, since robots could be programmed to serve our every need, the question arises of whether we would have less opportunity to cultivate this skill? Could this negatively impact how we relate to other people, in the sense that we may be less patient with others’ faults?

Human-robot relationships may not allow for ample opportunity to practise our moral skills. However, another concern is also that the way in which we morally conduct ourselves *within* these human-robot relations (i.e., how we treat these robots) may also impact the way in which we morally relate to other people. For example, arguing from a virtue ethical perspective, Sparrow (2017) argues that should we treat robots immorally, this would negatively impact our moral character. And Gerdes (2016) and Darling (2016), inspired by Immanuel Kant’s view on the moral treatment of animals, also argue that the way in which we treat robots, may impact the way in which we treat other people. Therefore, we should treat robots morally well (or should at least not treat them immorally).

What impact may treating robots morally *well* in these relations have upon the relations we have with other people? Sparrow (2021) argues that we cannot *actually* treat robots morally well (cf. Coeckelbergh, 2021). Aside from this, however, having what seems to be a meaningful and loving relationship with a robot may take us back to concerns about crowding out and potential moral deskilling, as discussed above. Therefore, humanoid robots may transform social and moral norms, in the context of when and how we relate to, and morally interact with, other people. Given that there may be certain negative implications that arise (as discussed above), this is something of which we should be aware.

### Human-Self Relations

More than just altering how we relate to technology and other people, humanoid robots may, further, alter the way in which we relate to ourselves, with regard to our understanding of what it means to be human. This is the third and, in the context of this paper, final way in which humanoid robots may transform norms surrounding our human relational experiences. This may occur in two ways: Firstly, we may question what it is that makes us unique, when we are confronted with the realisation that looking and behaving like a human being is no longer reserved for human beings alone. Thus, as humanoid robots become more sophisticated, we will continually search for defining aspects of humanity that set us apart. Secondly, more than motivating the quest for defining aspects of humanity, humanoid robots may also alter our understanding of human nature entirely, by inspiring a more mechanistic understanding of what it means to be human.

#### New Quests for Defining Aspects of Humanity

Human history is marked with ongoing philosophical discussions that attempt to answer the question “what does it mean to be human?” specifically with regard to thinking about what makes us so special compared to other species. Thus, philosophical literature makes reference to various defining aspects of humanity– such as politics, sociality, rationality, conscious thought, emotional capacities, moral capacities– that potentially characterise us as being particularly unique compared to other species. Defining what it is to be human, or thinking about what conditions must be fulfilled in order for the concept to be applicable, remains a philosophical challenge. However, the debate has new prominence today in light of certain smart technologies. As Sætra (2019, p. 66) notes: “intelligent machines are clearly a challenge to the belief that we are special, or in some way superior to (all) other things”.

For example, consider Aristotle’s well-known argument that humans are political (meaning sociopolitical) animals. If a robot can engage in sociopolitical activities, does this mean it is human? Sophia the robot, created by Hanson robotics, is probably one of the most famous humanoid robots. She, controversially, has been granted honourary Saudi Arabian citizenship and, moreover, has made appearances at a UN assembly meeting and Munich Security Conference (Sharkey, 2018; cf. Nyholm, 2020). Does her inclusion in such sociopolitical activities mean that we consider her human, in a sense? Most would be sceptical. As such, we may look for further conditions that distinguish us from a robot like Sophia.

Maybe it is our unique sense of morality that makes us special, given that we have the capacity to be both full moral agents and moral patients? This uniqueness may, too, be called into question as we see AI and robots that both look and behave like human beings. Regarding moral agency, the field of machine ethics seeks to create machines that can make moral decisions. In this regard, some argue that we already have machines that could, therefore, be considered moral agents, albeit in a limited sense (Floridi & Sanders, 2004; Sullins, 2006; List, 2021). However, as the field progresses, this may change. As for moral patiency, although current robots are not *real* moral patients, in the sense that they cannot feel what it is like to be treated in a certain way, some argue that what goes on “on the inside” does not matter as much as how the robot behaves. If it behaves as if it has moral patiency, we could consider it to have status as a moral patient or, at least, a perceived moral patient (Coeckelbergh, 2010; Danaher, 2020).

In prompting us to seek new defining aspects of humanity, humanoid robots may transform the way in which we understand and relate to ourselves, by challenging us to further question what it means to be human. Through coming face-to-face with an increasingly human-like technology, we may begin to doubt whether we are as unique as we like to think we are.

#### A Mechanistic Understanding of Humanity

More than moving the benchmark of what it is to be human, and causing us to question our human uniqueness, humanoid robots may cause us to have a more mechanistic understanding of what it means to be human. Thus, they not only force us to “search for other defining aspects of humanity” but might entirely change our understanding of humanity itself.

A mechanistic understanding of what it means to be human is nothing new, dating as far back as Hobbes’ notion of “the mechanization of mind” (Husbands et al., 2008). It can be related to the *synthetic method*: a method in which “artifacts are developed as models of human phenomena to gain knowledge of them” (Sætra, 2022, p. 7). More recently, we have seen how the brain is likened to a computer, leading to the influential computational theory of mind (Sætra, 2022), wherein “our minds are like computers implemented through neural activity” (ibid, p. 7).

However, with the development of humanoid robots, we see the synthetic method take place in a new age and context. Not only is the building of humanoid robots a recurring theme in science fiction (think Star Trek and Westworld, both of which use humanoid robots to explore what it means to be human) but it is also a “Holy Grail” for the AI community (Brooks et al., 1998), with Brooks et al. (1998, p. 52) stating that one of the reasons for building humanoid robots is to better understand “human cognition”. However, what is unique about humanoid robots is that they do not only mirror our brains, but through their embodiment, mirror our images too (Saætra, 2022).

Thus, one of the motivations behind the developments of humanoid robots, is to try and understand what it means to be human. Hanson (2017, p. 1)– founder of Hanson Robotics– shares this sentiment, stating that:

Humanlike robots reflect not just our faces but also our thoughts. They allow us to explore deep aspects about what makes us human by simulating human cognition, by testing people’s cognitive responses to the robots, and by challenging the human identity in robotic networks.

In a similar spirit, the roboticist Hiroshi Ishiguro, when being asked in an interview about why he began making humanoid robots, stated “My motivation for making humanoid robots stems from an interest in understanding what makes us human, and what it means to be human” (Barbican Centre, 2019).

However, in trying to understand what it means to be human through the creation of humanoid robots, a type of “reverse anthropomorphisation” (Jones, 2021) or “robotomorphy” (Sætra, 2022) may occur: we see something that looks so much like us, and behaves so much like us, yet we are acutely aware of its mechanical nature. We may, therefore, develop a more mechanistic understanding of what it means to be human. At first glance this may seem somewhat futuristic. However, we saw something similar occur when “scientists seeking to understand human (or animal) nature by studying rats proceed[ed] to transpose their findings to humans”, leading to some voicing concern that this created a warped understanding of human nature (Sætra, 2022, p. 8). Therefore, it is worthwhile to question whether the same could occur with humanoid robots. As we develop more sophisticated humanoid robots in order to understand ourselves better, could we transpose our findings to humans, thus developing a more mechanistic notion of what it means to be human? This is a second possible way in which humanoid robots may transform the way in which we relate to ourselves, by challenging and changing our understanding of what it means to be human.

## Conclusion

This paper explored the disruptive nature of humanoid robots. It elucidated upon how relating to humanoid robots as if they are human, yet being aware that they are not, may bring about changes to three types of human relational experiences: (1) the way we relate to technology; (2) the way we relate to other people; and (3) the way in which we relate to ourselves. It thus took the current debate surrounding the disruptiveness of humanoid robots beyond that of conceptual disruption, to also consider how humanoid robots transform and disrupt how we experience the world more broadly.

Elucidating upon the ways in which humanoid robots are disruptive, is one way in which to anticipate the ways in which they may impact and change society. Change, however, is not necessarily a bad thing. For example, our changing relations with technology, due to the tendency to relate to humanoid robots in social, emotional ways, may initially seem rationally misguided. However, this need not necessarily be perceived as a bad thing. Perhaps, it is indicative of our instinct to simply be kind:

“When people tell me that they feel for their [robot], I see people whose first instinct is to be kind. Our empathy is complex, self-serving, and sometimes incredibly misguided, but I’m not convinced that it’s a bad thing. Our hearts and brains don’t need to be opposed to each other…” (Darling, 2021).

In this regard, our tendency to relate to these robots in social and emotional ways (such as by perceiving them as being more human than tool, or even as moral patients) thereby leading to the disruption of our relations with technology, need not necessarily be cast in a negative light (Sect. 4.1). On the other hand, given concerns about social and emotional relations with robots crowding out human relations (as discussed in Sect. 4.2), we of course need to be aware that there may indeed be negative implications to relating to humanoid robots in these ways.

A complete analysis of the moral valence of the disruption of humanoid robots lies beyond the scope of this paper. However, that there are instances wherein this disruption may or may not be concerning, suggests that we should at least think carefully about the creation and proliferation of humanoid robots, before they become more sophisticated and widely used. Given this, future research may consider whether it is worth risking the negative impacts of the technology, for the value they bring. Or whether there are precautions we should consider so as to eliminate the risks they pose to society.

## Footnotes

1 As noted by Jones (2021, p. 2068): “The compulsion to create humanoid artefacts seems to have evolved with homo sapiens. Humanoid figurines carved from mammoth ivory were created between 40,000 and 35,000 years ago”.

2 http://www.miralab.ch/index.php/nadine-social-robot/.

3 https://eng.irl.sys.es.osaka-u.ac.jp/projects/erica.

4 https://spectrum.ieee.org/hiroshi-ishiguro-the-man-who-made-a-copy-of-himself.

5 https://www.hansonrobotics.com/sophia/.

6 http://www.parorobots.com/.

7 Some may be sceptical of this in light of the uncanny valley theory, put forward by Masahiro Mori in 1970. Mori proposed that realistic human-like robots would bring about a sense of unease or revulsion in people. Studies on the uncanny valley, however, have been inconsistent (Zhang et al., 2020), and some argue that “exposure to robots over time will reduce aspects of uncanniness, even in humanlike robots that are recognizable as artificial life” (Carpenter, 2017, p. 280; see also Kavner, 2012).
